# Clinical Characteristics and Real‐World Drug Treatment of Severe Pertussis in Children: A Retrospective Study

**DOI:** 10.1155/cjid/6702388

**Published:** 2025-12-12

**Authors:** Yangyang Wang, Jin Xu, Wenjing Li

**Affiliations:** ^1^ Department of Pharmacy, Jinhua Municipal Central Hospital, Jinhua, 321000, China, jhzxyy.cn; ^2^ Department of Pharmacy, Children’s Hospital of Nanjing Medical University, Nanjing, 210008, China, njmu.edu.cn

**Keywords:** children, drug treatment, economic evaluation, pertussis, severe, steroid treatment

## Abstract

**Background:**

Pertussis is an acute respiratory infectious disease caused by *Bordetella pertussis*, which can lead to respiratory failure and death in severe cases. In recent years, pertussis incidence has risen globally, especially in children, due to waning vaccine immunity and mutations in the pertussis bacteria. This study aimed to retrospectively analyze the clinical features, treatment regimens, and economic evaluation of pertussis in children to explore the impact of different treatments on disease outcomes.

**Methods:**

This study collected clinical data from 76 children with pertussis treated in our hospital from January 2024 to December 2024. The patients were divided into severe and nonsevere groups based on their condition. The clinical data, drug treatments, hospitalization costs, and hospital stays were compared between the two groups to evaluate the efficacy and economic impact of different treatments.

**Results:**

The study found that the highest incidence of pertussis occurred in infants aged 6 weeks to 3 months. Severe pertussis cases had significantly longer hospitalization times, treatment durations, and antibiotic therapy courses than the nonsevere group. Macrolide antibiotics remain the first‐line treatment, but resistance to these drugs has emerged in certain regions, making sulfamethoxazole–trimethoprim (SMZ‐TMP) an effective alternative. Steroid treatment was effective in symptom relief but did not affect cure rates or recurrence.

**Conclusion:**

This study shows that pertussis remains common in infants, with severe cases presenting greater treatment challenges. For children with macrolide‐resistant pertussis, SMZ‐TMP shows good efficacy. Proper selection of antibiotics and symptomatic treatment are essential for improving patient outcomes and reducing complications.

## 1. Introduction

Pertussis, also known as whooping cough, is a highly contagious acute respiratory disease caused by *Bordetella pertussis*. It is a vaccine‐preventable disease; however, despite the widespread implementation of vaccination in China, the incidence of pertussis has significantly decreased but has not been completely eradicated. Since the 1980s, several high‐vaccine‐coverage‐developed countries have witnessed a resurgence of pertussis, known as “pertussis resurgence” [1]. Similarly, China has encountered this public health challenge since 2011, with an increasing annual incidence rate. The reported incidence of pertussis in China from 2018 to 2022 ranged from 0.32 to 2.71 per 100,000 population, and in 2023, it increased to 2.89 per 100,000 population [2].

Although pertussis can affect individuals of all ages, it predominantly occurs in children, with significant variations in morbidity and mortality across different regions. According to the World Health Organization (WHO), approximately 2.24 million children under the age of 5 years are affected by pertussis annually, resulting in over 160,000 deaths, with a mortality rate of approximately 4% [3]. In China, the proportion of pertussis cases under 1 year old among the total number of cases has been reported to be as high as 31.17% [4], and infants are more prone to developing severe complications and progressing to severe pertussis. Severe pertussis in children may lead to critical complications such as respiratory failure, pertussis encephalopathy, and pulmonary hypertension (PH), significantly increasing the risk of mortality. Consequently, pertussis remains one of the leading causes of infection‐related mortality in children [3].

This study aimed to conduct a retrospective real‐world analysis of children diagnosed with severe pertussis who were hospitalized in our hospital from January 2024 to December 2024. We comprehensively evaluated the effectiveness, safety, and cost‐effectiveness of various drug treatments. Additionally, we explored the potential risk factors for disease progression to severe pertussis, aiming to provide clinical evidence for optimizing the treatment of severe pertussis in children and facilitating early prediction of disease progression in general cases.

## 2. Materials and Methods

### 2.1. Study Population

This study adopted a retrospective study design to collect and analyze the clinical data of children diagnosed with pertussis who were admitted to our hospital from January 2024 to December 2024. The study was approved by the Ethics Committee of Children’s Hospital of Nanjing Medical University, and informed consent was waived for all patients.

### 2.2. Diagnostic Criteria

All cases included in this study were laboratory‐confirmed according to the diagnostic criteria established by the Chinese Medical Doctor Association [5] and the National Health Commission of China [6]. A confirmed case was defined as one in which *Bordetella pertussis* was identified by culture, nucleic acid amplification testing, or next‐generation sequencing (NGS). Pertussis encephalopathy was defined, following the WHO [7] and National Health Commission of China [6] criteria, as acute toxic encephalopathy occurring during pertussis infection and presenting with neurological dysfunction such as convulsions, altered consciousness, or coma after exclusion of other potential causes. Hypoxemia was defined according to the WHO criteria as oxygen saturation (SpO_2_) < 90% [8]. Severe pertussis was defined as the presence of any of the following complications: recurrent apnea, hypoxemia, pertussis encephalopathy, or cardiovascular dysfunction [9].

### 2.3. Inclusion and Exclusion Criteria

Inclusion criteria were as follows: Patients meeting the confirmed cases and patients meeting severe pertussis criteria were classified into the severe pertussis group, whereas those without severe complications and those aged ≤ 18 years were categorized as the nonsevere pertussis group.

Exclusion criteria were as follows: Patients who did not meet the inclusion criteria and patients with incomplete clinical data were excluded;

for patients who were hospitalized multiple times during the study period, only the first hospitalization with a primary diagnosis of pertussis was included in the analysis.

### 2.4. Data Collection

Clinical data were retrieved from the hospital information system (HIS) database using diagnostic keywords, including pertussis, severe pertussis, pertussis encephalopathy, severe pneumonia, pertussis pneumonia, and bronchopneumonia. Cases that met the confirmed cases for pertussis were included in this study. The primary results of this study were the differences in general characteristics, clinical data, antibacterial agents’ treatment regimens, and clinical outcomes between the severe pertussis group and the nonsevere pertussis group. The secondary results were the differences in treatment regimens and clinical outcomes among the subgroups of age, vaccination status, underlying disease status, and coinfection, and the situation of cortisol treatment and blood exchange treatment in these children.

#### 2.4.1. General Information Collection

The collected general information included patient demographics (gender and age), admission date, and confirmed diagnosis before admission. Referring to the vaccination schedule and age distribution of pertussis onset reported in previous literature [10, 11], age was classified into six groups: < 6 weeks, 6 weeks to 3 months, 3 months–6 months, 6 months–12 months, 12 months to 3 years, and > 3 years.

#### 2.4.2. Clinical Data Collection

Clinical data were collected through a comprehensive review of patients’ medical records. The extracted information included admission symptoms, pertussis vaccination history, laboratory test results, presence of underlying diseases, coinfections, types and duration of medications, requirement for exchange transfusion, total hospitalization cost, drug‐related hospitalization cost, and length of hospital stay.

Clinical manifestations included facial flushing, cyanosis, whooping cough, wheezing, dyspnea, fever, retching or vomiting, dizziness or fatigue, and convulsions.

Laboratory data comprised routine hematological parameters, C‐reactive protein (CRP), procalcitonin (PCT), D‐dimer, and microbiological culture results. The microbiological data were analyzed for mixed infections, including coinfections with respiratory viruses, *Mycoplasma* pneumoniae, bacteria, or fungi. For bacterial isolates obtained from sputum, strict differentiation was made among contamination, colonization, and true infection. A case was classified as having bacterial superinfection when a positive culture was accompanied by elevated inflammatory markers (CRP and/or PCT), clinical features of infection (e.g., fever and tachypnea), or clinical and laboratory improvement following antibiotic therapy. Based on the characteristic hematological profile of pertussis [4], white blood cell (WBC) counts were stratified into six categories: ≤ 10 ∗ 10^9^/L, 10–15 ∗ 10^9^/L, 15–20 ∗ 10^9^/L, 20–30 ∗ 10^9^/L, 30–50 ∗ 10^9^/L, and > 50 ∗ 10^9^/L. Lymphocyte percentage was grouped into two categories: < 60% and ≥ 60%.

Antibiotic treatment was classified into five categories based on the initial and subsequent treatment regimens: macrolides alone (e.g., azithromycin and clarithromycin); sulfamethoxazole–trimethoprim (SMZ‐TMP) alone; beta‐lactam antibiotics containing *β*‐lactamase inhibitors (e.g., cefoperazone–sulbactam and piperacillin–tazobactam); initial treatment with one class of antibiotics followed by another; and initial treatment with one class of antibiotics, followed by another, and switched to a third antibiotic due to poor treatment response.

The use of corticosteroids during hospitalization was also recorded.

### 2.5. Statistical Analysis

Comparative analysis was conducted between the severe pertussis group and the nonsevere pertussis group regarding general characteristics, clinical data, treatment regimens, and clinical outcomes.

Statistical analysis was performed using SPSS 27.0. Continuous variables following a normal distribution were expressed as mean ± standard deviation ($\overset{¯}{x}$ ± *s*), whereas categorical variables were presented as frequency and percentage [*n* (%)]. The *t*‐test was used for comparisons of normally distributed continuous variables between groups, whereas categorical variables were analyzed using the chi‐square test (*X* ^2^ test). When there were cells with a theoretical frequency of less than 5 or the total sample size was less than 40, Fisher’s exact test was used instead. For the quantification of the association strength of categorical variables between groups, Cramer’s V coefficient is adopted as the effect size (with a value range of 0–1: Less than 0.3 is a weak association; 0.3–0.5 is a moderate association; and greater than 0.5 is a strong association) [12]. The test level was set at *α* = 0.05, and a *p*‐value < 0.05 was considered statistically significant.

## 3. Result

### 3.1. General Information

**Figure 1:**
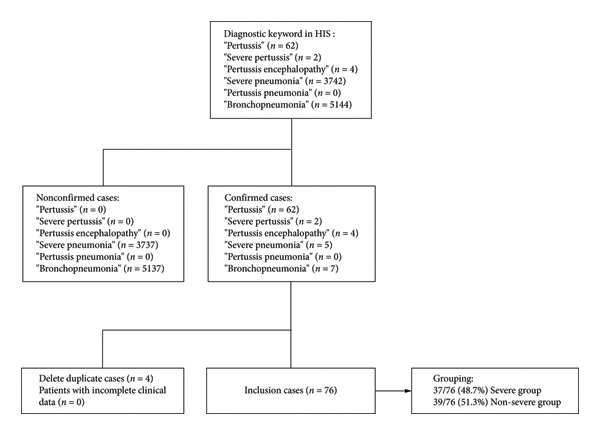
The flowchart of inclusion and exclusion criteria.

A total of 76 hospitalized children diagnosed with pertussis were included in this study, comprising 37 cases in the severe pertussis group and 39 cases in the nonsevere group (Figure 1).

The general characteristics of the patients are summarized in Table 1. Among the 76 patients, the male‐to‐female ratio was 1.375:1, and the median age was 3 months (range from 26 days to 11 years and 10 months). The 6‐week to 3‐month age group accounted for the largest proportion of cases (30 patients, 39.5%), followed by the 3‐ to 6‐month group (18 patients, 23.7%). Patients aged 6 weeks to 3 months had a significantly higher proportion of severe cases compared with the nonsevere group (*p* = 0.039, Cramer’s V = 0.237). In contrast, among children older than 3 years, the proportion of severe cases was significantly lower than that of the nonsevere group (*p* = 0.016, Cramer’s V = 0.277). When classified by season of admission, the highest proportion of cases occurred in spring (38.2%), with May accounting for the peak monthly incidence (17.11%), followed by February (11.84%). The lowest proportion of cases occurred in winter (18.4%). In the severe pertussis group, the proportion of cases occurring in winter was significantly higher than in the nonsevere group (*p* = 0.013, Cramer’s V = 0.284), whereas no significant differences were observed for other seasons. Overall, 47 cases (61.8%) were unvaccinated for pertussis, with a higher proportion in the severe group than in the nonsevere group (*p* = 0.631, Cramer’s V = 0.332). Only 14 cases (18.4%) had underlying medical conditions. Additionally, 51 cases (67.1%) had been diagnosed with *Bordetella pertussis* infection in outpatient clinics before hospitalization.

**Table 1:** General characteristics of 76 children with pertussis.

Variable	Total (*n* = 76)	Severe group (*n* = 37)	Nonsevere group (*n* = 39)	*p*‐Value
Gender, *n* (%)				
Male	44 (57.9)	18 (48.6)	26 (66.7)	0.112
Female	32 (42.1)	19 (51.4)	13 (33.3)	
*p*	0.169	0.869	0.037^∗^	—
Age, *n* (%)				
< 6 weeks	8 (10.5)	5 (13.5)	3 (7.7)	0.475
6 weeks–3 months	30 (39.5)	19 (51.4)	11 (28.2)	0.039^∗^
3–6 months	18 (23.7)	7 (18.9)	11 (28.2)	0.341
6–12 months	6 (7.9)	2 (5.4)	4 (10.3)	0.675
1–3 years	2 (2.6)	2 (5.4)	0 (0)	0.234
> 3 years	12 (15.8)	2 (5.4)	10 (25.6)	0.016^∗^
*p*	< 0.001^∗^	< 0.001^∗^	0.090	—
Seasonal distribution				
Spring (March–May)	29 (38.2)	14 (37.8)	15 (38.5)	0.955
Summer (June–August)	18 (23.7)	6 (16.2)	12 (30.8)	0.136
Autumn (September–November)	15 (19.7)	6 (16.2)	9 (23.1)	0.453
Winter (December–February)	14 (18.4)	11 (29.7)	3 (7.7)	0.013^∗^
*p*	0.058	0.168	0.044^∗^	—
Vaccination status				
Vaccinated	29 (38.2)	8 (21.6)	21 (53.8)	0.004^∗^
Unvaccinated	47 (61.8)	29 (78.4)	18 (46.2)	
*p*	0.039^∗^	0.001^∗^	0.631	—
Underlying conditions				
Present	14 (18.4)	7 (18.9)	7 (17.9)	0.913
Absent	62 (81.6)	30 (81.1)	32 (82.1)	
*p*	< 0.001^∗^	< 0.001^∗^	< 0.001^∗^	—
Prehospital pertussis diagnosis				
Diagnosed before admission	51 (67.1)	27 (73.0)	24 (61.5)	0.289
Diagnosed after admission	25 (32.9)	10 (27.0)	15 (38.5)	
*p*	0.003^∗^	0.005^∗^	0.150	—

^∗^ *p* < 0.05.

### 3.2. Clinical Symptoms

Upon admission, 60 of 76 children (78.9%) exhibited varying degrees of facial flushing or cyanosis. A total of 14 patients (18.4%) presented with whooping cough, whereas 11 patients (14.8%) experienced seizures, all of whom were in the severe group (*p* < 0.001, Cramer’s V = 0.422). Other symptoms, including wheezing, shortness of breath, fever, vomiting, dizziness, and fatigue, were observed at lower frequencies. The average duration of cough before admission was 13.5 ± 7.9 days. Detailed results are presented in Table 2.

**Table 2:** Clinical symptoms of 76 children with pertussis, *n* (%).

Clinical symptoms	Total (*n* = 76)	Severe group (*n* = 37)	Nonsevere group (*n* = 39)	*p*‐Value
Cyanosis/facial redness	60 (78.9)	29 (78.4)	31 (79.5)	0.906
Whooping cough	14 (18.4)	7 (18.9)	7 (17.9)	0.913
Seizures	11 (14.5)	11 (29.7)	0 (0)	< 0.001^∗^
Wheezing/shortness of breath	4 (5.3)	2 (5.4)	2 (5.1)	1.000
Fever	4 (5.3)	2 (5.4)	2 (5.1)	1.000
Vomiting	12 (15.8)	5 (13.5)	7 (17.9)	0.596
Dizziness/fatigue	1 (1.3)	1 (2.7)	0 (0)	0.487
Duration of cough before admission (d, $\overset{¯}{X}\pm S$)	13.53 ± 7.94	13.03 ± 6.73	14.03 ± 9.03	0.589

^∗^ *p* < 0.05.

### 3.3. Laboratory Test Results

The blood routine results of the 76 children, including WBC count, lymphocyte percentage, hemoglobin (Hb), platelet (PLT) count, CRP, PCT, and D‐dimer, were analyzed. The results are presented in Table 3.

**Table 3:** Laboratory test results of 76 children with pertussis.

Parameter	Total (*n* = 76)	Severe group (*n* = 37)	Nonsevere group (*n* = 39)	*p*‐Value
WBC count (^∗^10^9^/L, mean ± SD)	18.04 ± 11.21	22.07 ± 12.22	14.12 ± 8.60	0.002^∗^
WBC distribution, *n* (%)				
≤ 10 × 10^9^/L	18 (23.7)	4 (10.8)	14 (35.9)	0.010^∗^
10–15 × 10^9^/L	24 (31.6)	11 (29.7)	13 (33.3)	0.736
15–20 × 10^9^/L	8 (10.5)	4 (10.8)	4 (10.3)	0.937
20–30 × 10^9^/L	14 (18.4)	9 (24.3)	5 (12.8)	0.196
30–50 × 10^9^/L	10 (13.2)	8 (21.6)	2 (5.1)	0.044^∗^
> 50 × 10^9^/L	1 (1.3)	1 (2.7)	0 (0)	0.493
*p*	0.128	0.411	0.182	—
Lymphocyte percentage, *n* (%)				0.311
< 60%	43 (56.6)	21 (20.9)	22 (22.1)	—
≥ 60%	30 (39.5)	16 (14.6)	14 (15.4)	—
*p*	0.128	0.411	0.182	—
Hb (g/L, mean ± SD)	111.69 ± 13.99	107.05 ± 15.18	116.32 ± 11.05	0.004^∗^
PLT (^∗^10^9^/L, mean ± SD)	504.41 ± 192.77	564.08 ± 203.06	446.32 ± 164.85	0.007^∗^
CRP (mg/L, mean ± SD)	3.21 ± 13.32	5.9 ± 18.68	0.58 ± 0.99	0.092
PCT (ng/mL, mean ± SD)	0.13 ± 0.24	0.20 ± 0.33	0.06 ± 0.05	0.018^∗^
D‐dimer (mg/L, mean ± SD)	479.91 ± 1673.60	261.41 ± 151.31	708.81 ± 2398.00	0.387

^∗^ *p* < 0.05.

The mean WBC count among the 76 children was (18.04 ± 11.21) × 10^9^/L. The severe pertussis group had a significantly higher mean WBC count than the nonsevere group (*p* = 0.002). The proportion of patients with WBC ≤ 10 × 10^9^/L was significantly higher in the nonsevere group (*p* = 0.010, Cramer’s V = 0.295). As WBC levels increased, the difference between the two groups became less pronounced; however, when WBC exceeded 20 × 10^9^/L, the proportion of severe cases surpassed that of the nonsevere group. Notably, within the 30–50 × 10^9^/L range, the proportion of severe cases was significantly higher (*p* = 0.044, Cramer’s V = 0.244). Only one patient had a WBC > 50 × 10^9^/L, and this case belonged to the severe group. No statistically significant difference was found in the proportion of patients with lymphocyte percentage ≥ 60% between the two groups. The mean hemoglobin (Hb) level was significantly higher in the nonsevere group than in the severe group (*p* = 0.004). Among the 33 cases with abnormal Hb, 29 had mild anemia (20 in the severe group and 9 in the nonsevere group), whereas 4 had moderate anemia (3 in the severe group and 1 in the nonsevere group). PLT counts were elevated in 64 patients, equally distributed between the severe and nonsevere groups (32 cases each). However, the mean PLT count was significantly higher in the severe group (*p* = 0.007). Regarding inflammatory markers, six patients exhibited elevated CRP levels at admission, all of whom were in the severe group. Similarly, four patients had elevated PCT levels, also exclusively in the severe group. Consequently, the mean PCT level was significantly higher in the severe group than in the nonsevere group (*p* = 0.018).

### 3.4. Pathogen Examination Results

Among the 76 hospitalized children with pertussis, 41 cases (53.9%) involved single *Bordetella pertussis* infection, whereas 29 cases (38.1%) presented with mixed infections. Viral coinfections were the most frequent type, detected in 27.6% of patients. The most common viral pathogen was rhinovirus, accounting for 61.90% (13/21) of viral coinfections. In addition, seven children had bacterial coinfections, all of whom belonged to the severe pertussis group. The proportion of bacterial coinfections was significantly higher in the severe group than in the nonsevere group (*p* = 0.005, Cramer’s V = 0.327). Detailed data are summarized in Table 4.

**Table 4:** Pathogen examination results of 76 children with pertussis, *n* (%).

Infection type	Total (*n* = 76)	Severe group (*n* = 37)	Nonsevere group (*n* = 39)	*p*‐Value
Single *Bordetella pertussis* infection	41 (53.9)	20 (54.1)	27 (69.2)	0.173
Coinfection	29 (38.1)	17 (45.9)	12 (30.8)	
Viral infection	21 (27.6)	12 (30.8)	9 (24.3)	0.530
Bacterial infection	7 (9.2)	7 (18.9)	0 (0.0)	0.005^∗^
*Mycoplasma* infection	3 (3.9)	2 (5.4)	1 (2.6)	0.610
Fungal infection	1 (1.3)	1 (2.7)	0 (0.0)	0.487

^∗^ *p* < 0.05.

All bacterial coinfections involved the respiratory tract, diagnosed based on positive sputum culture results. Two cases were caused by *Enterobacter* spp., whereas four cases were attributed to *Streptococcus pneumoniae*, *Haemophilus influenzae*, *Pseudomonas aeruginosa*, and *Elizabethkingia meningoseptica*, respectively. One additional patient exhibited a concurrent central nervous system infection, with *Acinetobacter baumannii* detected in cerebrospinal fluid via NGS. Furthermore, three cases were identified as hospital‐acquired infections, characterized by negative sputum culture results upon admission but positive findings 24 h after admission.

### 3.5. Treatment Outcomes

An analysis was conducted on the length of hospital stay, PICU stay duration, duration of antimicrobial therapy, total hospitalization costs, medication costs, and average daily medication costs in the 76 pediatric patients. The results showed that severe cases had significantly longer hospital stays, PICU stays, and antimicrobial therapy durations compared to nonsevere cases (*p* < 0.05). Additionally, the total hospitalization costs and medication costs were significantly higher in the severe group than in the nonsevere group (*p* < 0.05). However, although the average daily medication cost was higher in severe cases, the difference was not statistically significant (*p* > 0.05). For detailed results, refer to Table 5.

**Table 5:** Treatment outcomes in 76 children with pertussis.

Variable	Total (*n* = 76)	Nonsevere group (*n* = 39)	Severe group (*n* = 37)	*p*
Length of hospital stay (days)	10.82 ± 7.77	7.03 ± 3.66	14.81 ± 8.93	< 0.001^∗^
PICU stay duration (days)	6.31 ± 8.71	1.82 ± 3.99	11.05 ± 9.82	< 0.001^∗^
Duration of antimicrobial therapy (days)	9.57 ± 7.07	6.36 ± 4.00	12.95 ± 8.02	< 0.001^∗^
Total hospitalization cost (10,000 RMB)	1.79 ± 2.05	0.84 ± 0.69	2.79 ± 2.50	< 0.001^∗^
Medication cost (10,000 RMB)	0.31 ± 0.52	0.13 ± 0.13	0.50 ± 0.69	0.003^∗^
Average daily medication cost (RMB)	260.53 ± 468.42	163.21 ± 150.45	363.12 ± 641.98	0.063

^∗^ *p* < 0.05.

#### 3.5.1. Antimicrobial Treatment

##### 3.5.1.1. Selection of Antimicrobial Agents

Among the 76 pediatric patients, 72 (94.7%) received antimicrobial treatment. Among those who received antibiotics, 45 patients (62.5%) were treated with a single antimicrobial agent for pertussis without switching medications. Of these, macrolide treatment accounted for the largest proportion (62.2%). The remaining 27 patients (37.5%) required a change in antimicrobial therapy during treatment. Additionally, eight patients (29.7%) sequentially received all three types of antibiotics, all of whom belonged to the severe group. In the nonsevere group, patients were more inclined to be treated with a single antimicrobial agent (*p* = 0.003, Cramer’s V = 0.352), and this tendency was even less pronounced in the severe group.

A subgroup analysis was conducted among the 72 patients who received antibiotic therapy, stratified by age, vaccination status, presence of underlying diseases, and coinfection status. The detailed findings are summarized in Table 6. The analysis revealed that the presence of bacterial coinfection was significantly associated with changes in antibiotic selection (*p* = 0.010, Cramer’s V = 0.327), suggesting that bacterial infection may be an important factor influencing clinicians’ antibiotic choice.

**Table 6:** Selection of antimicrobial agents in 72 children with pertussis, *n* (%).

Category	Single‐agent therapy (*n* = 45)				Changed antibiotic therapy (*n* = 27)					*p*
	M	S	B	*p*	M‐S	M‐B	S‐B	M‐S‐B	*p*	
Total (*n* = 72)	28 (62.2)	7 (15.6)	10 (22.2)	< 0.001^∗^	6 (22.2)	10 (37.0)	3 (11.1)	8 (29.6)	0.265	0.034^∗^
Disease severity										0.003^∗^
Severe group (*n* = 37)	9 (52.9)	4 (23.5)	4 (23.5)	0.230	3 (15.0)	7 (35.0)	2 (10.0)	8 (40.0)	0.158	—
Nonsevere group (*n* = 35)	19 (67.9)	3 (10.7)	6 (21.4)	< 0.001^∗^	3 (42.9)	3 (42.9)	1 (14.3)	0 (0.0)	0.565	—
*p*	0.317	0.399	0.869	—	0.290	0.711	0.756	0.068	—	—
Age group										0.301
≤ 3 Months (*n* = 37)	13 (61.9)	4 (19.0)	4 (19.0)	0.021^∗^	1 (6.3)	6 (37.5)	2 (12.5)	7 (43.8)	0.090	—
> 3 Months (*n* = 35)	15 (62.5)	3 (12.5)	6 (25.0)	0.008^∗^	5 (45.5)	4 (36.4)	1 (9.1)	1 (9.1)	0.200	—
*p*	0.967	0.689	0.729	—	0.027^∗^	0.952	0.782	0.090	—	—
Vaccination status										0.285
Vaccinated (*n* = 27)	12 (63.2)	2 (10.5)	5 (26.3)	0.016^∗^	4 (50.0)	3 (37.5)	1 (12.5)	0 (0)	0.417	—
Unvaccinated (*n* = 45)	16 (61.5)	5 (19.2)	5 (19.2)	0.008^∗^	2 (10.5)	7 (36.8)	2 (10.5)	8 (42.1)	0.091	—
*p*	0.912	0.681	0.720	—	0.044^∗^	0.974	0.882	0.061	—	—
Presence of underlying disease										0.214
With underlying disease (*n* = 13)	6 (100.0)	0 (0)	0 (0)	—	1 (14.3)	2 (28.6)	1 (14.3)	3 (42.9)	0.666	—
Without underlying disease (*n* = 59)	22 (56.4)	7 (17.9)	10 (25.6)	0.008^∗^	5 (25.0)	8 (40.0)	2 (10.0)	5 (25.0)	0.308	—
*p*	0.069	0.569	0.312	—	0.557	0.678	0.756	0.633	—	—
Coinfection status										0.010^∗^
With bacterial coinfection (*n* = 7)	0 (0)	1 (100)	0 (0)	—	2 (33.3)	2 (33.3)	0 (0)	2 (33.3)	1.000	—
Without bacterial coinfection (*n* = 65)	28 (63.6)	6 (13.6)	10 (22.7)	< 0.001^∗^	4 (19.0)	8 (38.1)	3 (14.3)	6 (28.6)	0.467	—
*p*	0.378	0.156	1.000	—	0.588	1.000	1.000	1.000	—	—

*Note:* M, macrolides; S, sulfamethoxazole–trimethoprim; B, beta‐lactam/beta‐lactamase inhibitor combinations; M‐S, switch from macrolides to sulfamethoxazole–trimethoprim; M‐B, switch from macrolides to beta‐lactam/beta‐lactamase inhibitor combinations; S‐B, switch from sulfamethoxazole–trimethoprim to beta‐lactam/beta‐lactamase inhibitor combinations; M‐S‐B, switch from macrolides to sulfamethoxazole–trimethoprim and then to beta‐lactam/beta‐lactamase inhibitor combinations.

^∗^ *p* < 0.05.

##### 3.5.1.2. Antimicrobial Treatment Outcomes

An analysis was conducted on the hospitalization time, PICU stay duration, antimicrobial treatment course, hospitalization costs, medication treatment costs, and daily drug expenses of 72 children treated with antimicrobial agents. The results showed that among the children who received a single antimicrobial agent for pertussis treatment (without switching), those treated with *β*‐lactam antibiotics containing *β*‐lactamase inhibitors had the shortest course, hospitalization time, and PICU stay duration. This was followed by children treated with macrolides and those treated with SMZ‐TMP.

Among the children who switched antimicrobial agents during treatment, those who initially received macrolides and then switched to SMZ‐TMP had the shortest course, hospitalization time, and PICU stay duration, followed by those who initially received macrolides and then switched to *β*‐lactam antibiotics containing *β*‐lactamase inhibitors. Those who received three different types of antibiotics sequentially had the longest course, hospitalization time, and PICU stay duration.

In terms of costs, among the children who received a single antimicrobial agent for pertussis treatment (without switching), those treated with SMZ‐TMP had the highest hospitalization costs. Children treated with *β*‐lactam antibiotics containing *β*‐lactamase inhibitors had lower hospitalization costs compared to the other two groups. Regarding medication treatment costs and daily drug expenses, children treated with *β*‐lactam antibiotics containing *β*‐lactamase inhibitors had the highest medication costs, whereas those treated with SMZ‐TMP had lower medication costs compared to the other two groups. Among the children who switched antimicrobial agents during treatment, those who sequentially used three different types of antimicrobial agents had higher hospitalization costs, medication treatment costs, and daily drug expenses compared to the other three treatment strategies. Conversely, children who initially received macrolides and then switched to SMZ‐TMP had the lowest hospitalization costs, medication treatment costs, and daily drug expenses. The results are shown in Table 7.

**Table 7:** Comparison of treatment outcome among different antibiotic regimens, mean ± SD.

Variable	Single‐agent therapy (*n* = 45)			Changed antibiotic therapy (*n* = 27)			
	M (*n* = 28)	S (*n* = 7)	B (*n* = 10)	M‐S (*n* = 6)	M‐B (*n* = 10)	S‐B (*n* = 3)	M‐S‐B (*n* = 8)
Duration of antimicrobial therapy (days)	7.54 ± 5.52	9.29 ± 3.09	7.00 ± 2.26	9.50 ± 6.66	13.00 ± 7.17	17.33 ± 11.06	17.75 ± 8.55
Length of hospital stay (days)	8.89 ± 6.32	10.14 ± 2.91	7.90 ± 2.28	10.33 ± 8.57	15.20 ± 9.78	17.33 ± 11.06	18.38 ± 9.30
PICU stay duration (days)	4.25 ± 7.70	6.86 ± 3.58	0.00 ± 0.00	5.33 ± 8.14	12.10 ± 11.40	12.33 ± 12.50	14.50 ± 9.09
Total hospitalization cost (10,000 RMB)	1.27 ± 1.31	1.36 ± 0.45	0.87 ± 0.38	2.05 ± 2.79	2.90 ± 2.87	2.61 ± 1.72	4.01 ± 3.07
Medication cost (10,000 RMB)	0.18 ± 0.21	0.12 ± 0.06	0.20 ± 0.13	0.33 ± 0.63	0.50 ± 0.49	0.53 ± 0.22	0.91 ± 1.21
Daily medication cost (RMB)	170.11 ± 158.79	126.00 ± 92.09	250.12 ± 155.2	187.84 ± 206.14	287.96 ± 153.77	336.85 ± 82.31	806.04 ± 1314.27

*Note:* M, macrolides; S, sulfamethoxazole–trimethoprim; B, beta‐lactam/beta‐lactamase inhibitor combinations; M‐S, switch from macrolides to sulfamethoxazole–trimethoprim; M‐B, switch from macrolides to beta‐lactam/beta‐lactamase inhibitor combinations; S‐B, switch from sulfamethoxazole–trimethoprim to beta‐lactam/beta‐lactamase inhibitor combinations; M‐S‐B, switch from macrolides to sulfamethoxazole–trimethoprim and then to beta‐lactam/beta‐lactamase inhibitor combinations.

#### 3.5.2. Corticosteroid Therapy

Among the 76 patients, 58 patients (76.3%) received corticosteroid therapy during treatment, and the average course of treatment was 6.47 ± 3.89 days. There was no statistically significant difference between the severe group and the nonsevere group regarding corticosteroid use (*p* = 0.341). A subgroup analysis was conducted based on age distribution (divided into the group > 3 months old and the group ≤ 3 months old), vaccination status, presence of underlying diseases, and coinfections. The results showed no significant differences in corticosteroid use across these subgroups (*p* > 0.05).

Compared to children who did not receive corticosteroid treatment, there were no statistically significant differences in hospitalization time, PICU stay duration, antibiotic treatment course, hospitalization costs, medication costs, and average daily medication costs in the children who received corticosteroid treatment (*p* > 0.05).

#### 3.5.3. Exchange Blood Transfusion (EBT)

Among the 76 patients, five children (6.58%) underwent EBT, all of whom were classified in the severe pertussis group. Their ages were below 3 months, with the youngest being 26 days old. The female‐to‐male ratio was 4:1, and none of the five children had received pertussis vaccination. Three patients were diagnosed at outside hospitals and subsequently transferred to our hospital. The mean duration of cough before admission was 12.40 ± 6.41 days, convulsions occurred in three cases, and two had underlying congenital heart disease. Before EBT, the mean WBC count was 43.07 ± 6.73 × 10^9^/L, with a maximum value of 55.71 × 10^9^/L. Following EBT, the WBC count decreased to 21.97 ± 4.83 × 10^9^/L, representing an average reduction of 21.10 ± 6.65 × 10^9^/L. During hospitalization, three patients developed respiratory failure, three developed pertussis encephalopathy, and two developed cardiac insufficiency. One death occurred in a 26‐day‐old infant who presented with concurrent central nervous system infection (*Acinetobacter baumannii*), respiratory failure, pertussis encephalopathy, and cardiac insufficiency.

## 4. Discussion

Pertussis is classified as a Category B infectious disease in China and is preventable through vaccination. Since the introduction of the diphtheria–tetanus–whole‐cell pertussis (DTwP) vaccine into the national immunization program in 1978, the incidence and mortality rates of pertussis have significantly declined. Between 2007 and 2012, DTwP was gradually replaced by the diphtheria–tetanus–acellular pertussis (DTaP) vaccine [13]. However, in recent years, there has been a resurgence in pertussis cases, potentially due to waning vaccine‐induced immunity and genetic variations in *Bordetella pertussis*. This phenomenon, known as “pertussis resurgence,” has been observed in many countries with high vaccine coverage, including China. Moreover, the peak age of infection has shifted from infants to adolescents and adults, who now serve as the primary source of transmission to infants. Understanding the epidemiological characteristics of this pertussis resurgence—particularly its clinical manifestations and treatment strategies in pediatric populations—is essential for optimizing the diagnosis, treatment, and prevention of pertussis in children. This study retrospectively analyzed the clinical data of 76 pediatric confirmed cases with pertussis who were admitted to our hospital between January and December 2024. The diagnostic definitions in the screening process of these cases were based on guidelines for diagnosis and management and prevention of pertussis of China (2024 edition) compiled by the Chinese Medical Association and Diagnosis and treatment plan for pertussis (2023 edition) compiled by the National Health Commission of China (with roughly the same framework as WHO). Based on clinical diagnosis, laboratory nucleic acid testing, culture, or NGS positive (the antibodies mentioned in the guidelines have not been tested in our hospital and are therefore not applicable to this study), the patients were categorized into the severe and nonsevere groups based on disease severity. By comparing demographic characteristics, clinical features, pharmacological treatments, clinical outcomes, and economic burden, this study aims to provide valuable insights for the management of pediatric pertussis and the prevention of severe cases.

The highest proportion of cases occurred in infants aged 6 weeks to 3 months (30 cases, 39.5%) in this study, followed by those aged 3–6 months (18 cases, 23.7%). This age distribution may be attributed to the fact that maternally derived antibodies have a half‐life of only 6 weeks after birth, gradually losing their protective effect as they wane [14, 15]. Additionally, according to China’s national immunization schedule, children receive the DTaP vaccine at 3, 4, and 5 months of age, with a booster dose at 18 months [16, 17]. As a result, infants aged 6 weeks to 3 months lack vaccine‐induced protection, making them more susceptible to infection. Furthermore, the immaturity of the immune system in younger infants may also contribute to their higher susceptibility to pertussis [18]. Previous studies have shown that the hospitalization rate, complication rate, and mortality rate of pertussis are highest among infants compared to other age groups [19]. Severe pertussis is particularly prevalent in infants younger than 3 months, with a mortality rate as high as 34.2%. The majority of fatalities occur in infants younger than 6 weeks [20]. These findings align with our study results, which showed a significantly higher proportion of severe cases in the 6‐week to 3‐month age group. The seasonal distribution of pertussis may vary around the world [21–24]. In a study focusing on Beijing, China [11], pertussis cases are most prevalent in summer (38.2%) and least in winter (15.5%), but in our research, the highest proportion of cases occurred in spring (38.2%), followed by summer (23.7%), autumn (19.7%), and winter (18.4%). The two studies show differences in the high‐incidence seasons of cases. The hospital where this research is conducted is located in southern China. In China, the south generally enters summer earlier than the north, which might be the main reason for the difference. Moreover, the high‐incidence month of pertussis in this study is May (17.11%), which falls within the period when spring and summer overlap and further supports the speculation about the main cause. The reason why there are the fewest cases in winter might be that other respiratory pathogens, including viruses and bacteria, are very active in winter. The coinfection of pertussis with other pathogens may complicate bacterial isolation, which suggests that improving testing techniques can prevent the missed diagnosis of pertussis in mixed infections. Vaccination has been shown to effectively prevent pertussis, significantly shorten the disease duration in severe cases, and reduce the frequency of paroxysmal coughing episodes [25]. This explains why, in our study, the proportion of unvaccinated children in the severe group was significantly higher than that of vaccinated children. Although the presence of underlying medical conditions did not significantly affect the incidence of severe versus nonsevere pertussis, it may influence vaccine administration in some children with comorbidities.

The typical clinical manifestations of pertussis include paroxysmal spasmodic coughing, often accompanied by an inspiratory whooping sound at the end of a coughing episode, as well as peripheral blood lymphocytosis [26]. In this study, 78.9% of the patients exhibited facial flushing, cyanosis, or dyspnea, whereas only 18.4% presented with the characteristic whooping cough. These findings are consistent with previous studies [10], highlighting the importance of early laboratory testing—such as polymerase chain reaction (PCR) analysis of respiratory secretions—for timely diagnosis and treatment of suspected cases. Additionally, 11 patients (14.8%) experienced seizures upon hospital admission, all of whom were diagnosed with pertussis encephalopathy. Neurological symptoms may persist after recovery, underscoring the need for clinicians to differentiate pertussis‐related neurological complications from other central nervous system disorders.

In the early stages of pertussis, patients often exhibit a significant increase in peripheral blood leukocyte count, which can reach (20–50) × 10^9^/L or even exceed 70 × 10^9^/L. Elevated leukocyte levels have been identified as an independent risk factor for severe pertussis [27–29]. Excessively high leukocyte counts, combined with reduced deformability, can slow pulmonary blood flow, increasing the risk of embolism in the narrow alveolar capillary beds, which may lead to PH, hypoxemia, and even heart failure—common complications of severe pertussis. In this study, 75.1% of patients exhibited elevated leukocyte counts, with 31.6% having counts in the range of (20–50) × 10^9^/L and one patient reaching 54.33 × 10^9^/L. The mean leukocyte count in the severe group was significantly higher than in the nonsevere group (*p* = 0.002). Moreover, as leukocyte counts increased, the proportion of nonsevere and severe cases became more comparable. When leukocyte counts exceeded 20 × 10^9^/L, the proportion of severe cases surpassed that of nonsevere cases, and at 30–50 × 10^9^/L, the proportion of severe cases was significantly higher. Additionally, pertussis toxin promotes the release of lymphocytes from peripheral blood reservoirs into circulation, often increasing lymphocyte percentages to 60%–90%. This phenomenon is more commonly observed in unvaccinated infants but is relatively less frequent in older children and vaccinated individuals. However, this study did not observe a significant increase in lymphocyte ratio. Other laboratory findings showed that the mean hemoglobin levels were higher in the nonsevere group than in the severe group, whereas the severe group had higher mean PLT counts, CRP, and PCT levels compared to the nonsevere group. Based on past experience, the abnormalities of these indicators may not have a direct connection with pertussis infection. However, in clinical treatment, they remain a focus of our attention. On the one hand, these indicators can guide clinical medication, and on the other hand, they can assist in prognosis analysis in clinical practice.

In this study, the sputum culture positivity rate among hospitalized children with pertussis was 36.84% (28/76), increasing to 54.05% in the severe group. After excluding cases of possible bacterial colonization and including only those with confirmed bacterial infections, 18.9% of patients in the severe group were identified as having bacterial coinfections, a proportion significantly higher than that observed in the nonsevere group. Coinfection with other pathogens has been shown to exacerbate disease severity in pertussis. In our cohort, patients with bacterial or viral coinfections were more likely to develop pulmonary consolidation and respiratory failure, which are associated with increased clinical complexity and longer hospital stays. These findings underscore the importance of comprehensive pathogen screening during hospitalization and the need for timely, targeted antimicrobial therapy to prevent further deterioration and improve clinical outcomes.

In our cohort, antibiotic therapy was primarily guided by national and international pertussis treatment guidelines, which recommend macrolides as the first‐line agents [17]. Among the 72 children who received antimicrobial therapy, 72.22% were initially treated with macrolides. However, in real‐world clinical practice, several factors contributed to deviations from guideline‐based recommendations. First, the antimicrobial susceptibility of *Bordetella pertussis* plays a critical role in antibiotic selection. Recent studies have reported that the macrolide resistance rate of *B. pertussis* isolates in certain regions of China has reached 70%–100% [30, 31], resulting in poor bacterial clearance rates for erythromycin and azithromycin against resistant strains [32]. SMZ‐TMP has demonstrated a minimum inhibitory concentration (MIC90) of 0.3 mg/L against *B. pertussis* isolates [33, 34], with treatment efficacy and clearance rates comparable to erythromycin [35]. Therefore, SMZ‐TMP is recommended as the first‐line alternative in cases of macrolide‐resistant pertussis or treatment failure in patients aged over 2 months. Second, patient‐specific factors such as being younger than 2 months or having cardiovascular comorbidities also influenced antibiotic selection due to safety concerns. Observational studies suggested that piperacillin–tazobactam and cefoperazone–sulbactam may be more effective than erythromycin or azithromycin for pertussis treatment in China [32]. Consequently, for patients in whom SMZ‐TMP is contraindicated, such as those younger than 2 months, or with glucose‐6‐phosphate dehydrogenase (G6PD) deficiency, severe allergies, or renal impairment, piperacillin–tazobactam or cefoperazone–sulbactam may serve as suitable alternatives. Levofloxacin, due to its limited pediatric use, is reserved for adult patients. In our study, among patients who required antibiotic modification due to treatment failure, most switched from macrolides to *β*‐lactam/β‐lactamase inhibitor combinations. This likely reflects the high proportion of infants under 2 months in our cohort, for whom SMZ‐TMP is contraindicated. Additionally, the presence of bacterial coinfections prompted clinicians to select broader‐spectrum agents guided by susceptibility testing. Economic considerations occasionally influenced drug choice, with SMZ‐TMP being preferred for its lower cost (as demonstrated in the cost analysis section of this study). These variations reflect real‐world clinical decision‐making but may introduce bias in analyses of antibiotic use and hospitalization costs. As this study was retrospective, antibiotic selection was based on documented clinical practice, and the inherent limitations of medical record data should be acknowledged. Nevertheless, our findings provide valuable insight into the practical challenges of pertussis management and underscore the importance of individualized, guideline‐informed therapy within the framework of antimicrobial stewardship.

Compared to patients who did not receive glucocorticoid therapy, those treated with glucocorticoids experienced a reduction in symptom duration, and no steroid‐related adverse effects were observed during follow‐up. However, glucocorticoid therapy did not influence the treatment efficacy, recurrence rate, or incidence of post–treatment complications (such as pneumonia) in pertussis patients [36, 37]. Therefore, for patients with typical pertussis experiencing paroxysmal spasmodic coughing or repeated episodes of apnea, a short course of systemic glucocorticoid therapy (no longer than 7–10 days) may be considered. However, systemic glucocorticoid therapy can lead to a significant increase in peripheral blood leukocyte counts (≥ 30 × 10^9^/L), which may contribute to PH and cardiopulmonary dysfunction. Thus, in severe pertussis cases, if leukocyte counts are already markedly elevated and there is a risk of PH, cardiopulmonary dysfunction, or the need for mechanical ventilation, glucocorticoid therapy should be used with caution [38]. In this study, the proportion of glucocorticoid use was lower in the severe group than in the nonsevere group, though the difference was not statistically significant. Additionally, there was no significant association between glucocorticoid use and factors such as age distribution, vaccination status, presence of underlying diseases, or concurrent infections.

Death in young infants with severe pertussis is typically linked to profound leukocytosis with lymphocytosis, refractory PH, and cardiogenic shock [39]. In cases of leukocytosis with lymphocytosis, EBT may be considered [5]. EBT has been reported to enhance outcomes in patients with severe pertussis who experience life‐threatening complications that persist despite aggressive therapeutic interventions [40]. In our study, five infants with elevated WBCs underwent EBT. None of them had reached the time for vaccination and developed severe complications after infection. Regrettably, even after EBT, there was still one child who died in an extremely short period of time. Some severe cases may also require extracorporeal membrane oxygenation (ECMO). In our study, although ECMO technology is available in our hospital, none of the included patients received ECMO. This may contribute to differences in outcomes compared with centers where ECMO is more frequently applied. Future studies should consider ECMO use to further evaluate its efficacy and safety in critically ill children with pertussis.

The length of hospital stay, duration of PICU admission, and total course of antibiotic treatment were longer in the severe group than in the nonsevere group. Additionally, the total hospitalization costs and medication expenses were higher in the severe group, although there was no significant difference in the average daily medication cost. A possible explanation for this is that severe pertussis often requires more intensive supportive management, including mechanical ventilation, inhaled nitric oxide (NO), and EBT. In critical cases, ECMO may also be necessary. As a result, the increased costs are primarily driven by nonpharmacological interventions rather than medication expenses.

## 5. Limitation

This study has several limitations. Firstly, as a single‐center study with a relatively small sample size, the findings may not be generalizable to broader populations. To minimize bias associated with the small sample, we applied Fisher’s exact test and calculated the effect size to enhance the robustness of the analysis. Second, due to the retrospective nature of the study, the accuracy of certain data may have been affected by the quality of medical record documentation, particularly regarding the incidence of symptoms at admission, which might have been underestimated. Third, long‐term outcomes following treatment were not assessed, limiting our ability to evaluate the sustained efficacy and prognosis of different therapeutic approaches. Finally, the lack of quantitative bacterial load data and antimicrobial susceptibility testing required us to rely on retrospective clinical information to distinguish bacterial colonization from true infection. Moving forward, we plan to build upon this research by conducting a multicenter, longitudinal study involving diverse geographic regions across China. Such an approach will enable a more comprehensive investigation of severe pertussis and its treatment strategies, with the goal of identifying broader and more effective therapeutic options for pediatric pertussis in clinical practice.

## 6. Conclusion

This retrospective study analyzed the clinical data of 76 children with pertussis, revealing that pertussis remains relatively common among the pediatric population, particularly in infants aged 6 weeks to 3 months. Severe pertussis cases were associated with longer hospital stays, longer antibiotic treatment courses, and higher treatment costs, with a higher incidence of bacterial coinfections. Macrolide antibiotics remain the first‐line treatment, but resistance to these drugs has been reported in some regions. Therefore, SMZ‐TMP shows good efficacy as an alternative treatment for macrolide‐resistant cases. Steroid treatment is effective in symptom relief but does not impact cure rates or recurrence. For severe pertussis cases, especially those with coinfections or requiring supportive treatment, a comprehensive approach to therapy is necessary to improve outcomes and reduce complications.

## Ethics Statement

All procedures followed were in accordance with the ethical standards of the responsible committee on human experimentation (institutional and national) and the Helsinki Declaration of 1964 and later versions. The study was approved by the Ethics Committee of Children’s Hospital of Nanjing Medical University (approval number 202510040‐1), and informed consent from patients was waived.

## Consent

Please see Ethics Statement.

## Conflicts of Interest

The authors declare no conflicts of interest.

## Author Contributions

Dr Yangyang Wang was responsible for the design of the research protocol, data collection, and analysis, drafted the initial manuscript, and revised and confirmed the final version of the manuscript. Dr Jin Xu provided technical support required for the study, participated in the optimization of the research protocol, offered revision suggestions on the core viewpoints of the manuscript, and approved the final published version. Dr Wenjing Li oversaw the research project, guided the experimental design and data interpretation, and conducted a critical review of the important intellectual content in the manuscript.

## Funding

The authors received no specific funding for this work.

## Data Availability

The data that support the findings of this study are available from the corresponding author upon reasonable request. The data are not publicly available due to privacy or ethical restrictions.
